# Immunostimulatory Effects of Live *Lactobacillus sakei* K040706 on the CYP-Induced Immunosuppression Mouse Model

**DOI:** 10.3390/nu12113573

**Published:** 2020-11-22

**Authors:** Seo-Yeon Kim, Ji-Sun Shin, Kyung-Sook Chung, Hee-Soo Han, Hwi-Ho Lee, Jeong-Hun Lee, Su-Yeon Kim, Yong Woo Ji, Yejin Ha, Jooyeon Kang, Young Kyoung Rhee, Kyung-Tae Lee

**Affiliations:** 1 Department of Pharmaceutical Biochemistry, College of Pharmacy Kyung Hee University, Seoul 02447, Korea; tjdus7772@hanmail.net (S.-Y.K.); jsunvet@naver.com (J.-S.S.); adella76@hanmail.net (K.-S.C.); heesu3620@daum.net (H.-S.H.); hhlee4083@naver.com (H.-H.L.); ztztzt08@hanmail.net (J.-H.L.); dlstm4@gmail.com (S.-Y.K.); 2 Department of Life and Nanopharmaceutical Sciences, Graduate School, Kyung Hee University, Seoul 02447, Korea; 3 Department of Ophthalmology, National Health Insurance Service Ilsan Hospital, Goyang 10444, Korea; lusita30@gmail.com; 4 Institute of Vision Research, Department of Ophthalmology, Yonsei University College of Medicine, Seoul 03722, Korea; 5 NOVAREX Co. Ltd., 94, Gangni 1-gil, Ochang-eup, Cheongwon-gu, Cheongju-si, Chungcheongbuk-do 363-885, Korea; yj9113@novarex.co.kr (Y.H.); kjy@novarex.co.kr (J.K.); 6 Korea Food Research Institute, Wanju-gun, Jeollabuk-do 55365, Korea; ykrhee@kfri.re.kr

**Keywords:** *Lactobacillus sakei*, immunosuppression, NK cell, cyclophosphamide, microbiota, splenocyte, Peyer’s patch

## Abstract

Our previous studies have shown that heat-killed *Lactobacillus sakei* K040706 exerts immunostimulatory and anti-inflammatory activities in macrophages, cyclophosphamide (CYP)-treated mice, and dextran sulfate sodium–induced colitis mice. However, the immunostimulatory effects of live *Lactobacillus sakei* K040706 (live K040706) against CYP-induced immunosuppression and its underlying molecular mechanisms remain unknown. Therefore, we investigated the immunostimulatory effects of live K040706 (10^8^ or 10^9^ colony forming unit (CFU)/day, p.o.) in CYP-induced immunosuppressed mice. Oral administration of live K040706 prevented the CYP-induced decreases in body weight, thymus index, natural killer (NK) cell activity, T and B cell proliferation, and cytokine (interferon (IFN)-γ, interleukin (IL)-2, and IL-12) production. The administration of live K040706 also exerted positive effects on the gut microbiota of CYP-induced mice, resulting in a microbiota composition similar to that of normal mice. Moreover, live K040706 significantly enhanced IL-6 and granulocyte-macrophage colony-stimulating factor (GM-CSF) production in the splenocytes and Peyer’s patch (PP) cells of mice and increased bone marrow (BM) cell proliferation. Taken together, our data indicate that live K040706 may effectively accelerate recovery from CYP-induced immunosuppression, leading to activation of the immune system. Therefore, live K040706 may serve as a potential immunomodulatory agent against immunosuppression.

## 1. Introduction

Immunomodulators, known as biological response modifiers, enhance the host defense system by upregulating or downregulating components of the nonspecific immune system, including granulocytes, macrophages, complements, certain T lymphocytes, natural killer (NK) cells, and different effector substances [1]. They can contribute to immune defense by activating the innate immune system, enhancing cell-mediated immunity to fight established infections, and attacking cancer cells via cytokines, tumor-specific antibodies, and tumor-penetrating lymphocytes [2]. Among them, T helper (Th) cells play crucial roles in regulating the known immune responses [3]. Type 1 T helper (Th1) cells produce interferon-gamma (IFN-γ), interleukin (IL)-2, IL-12, and tumor necrosis factor (TNF)-β, which activate the macrophages and are responsible for phagocyte-dependent protective responses, thus overcoming intracellular infections by viruses and bacteria [4]. Type 2 T helper (Th2) cells produce IL-4, IL-5, IL-10, and IL-13, which are responsible for strong antibody production, B cell proliferation, eosinophil activation, and phagocyte-independent protective responses; thus, these cells are necessary for protection against extracellular parasites, bacteria, allergens, and toxins [4,5].

Being natural immunomodulatory agents, probiotics are live microorganisms that can confer health benefits to the host. Their effects can be divided into three main categories (metabolic, protective, and tropic), and they stimulate various functions, including the reinforcement and regulation of the immune system and the prevention of pathogen infringement [6]. Probiotics function in the intestines by controlling the microbiota, immune parameters, and intestinal permeability; however, they also affect other organs by producing bioactive or regulated metabolites [6]. Several studies have shown that certain probiotic strains can exert potent immunomodulatory activity through their interaction with a diverse range of immune cells (lymphocytes, NK cells, and antigen-presenting cells) in various disorders, including allergic asthma and inflammatory bowel diseases. This interaction could contribute to the maintenance of immune homeostasis, thereby stabilizing the immune response [7,8].

Lactic acid bacteria (LAB), which are members of the order Lactobacillales, are typical probiotic bacteria. LAB are commonly found in yogurt, kimchi, cheese, and the vaginal tracts of humans and animals. Previous studies have suggested that LAB can exert positive effects on digestion and the immune system [8,9]. *Lactobacilli sakei* K040706 (K040706) are found in the traditional Korean fermented food Doenjang, which is prepared by fermenting moldy cooked soybeans (Meju) in brine. This process results in the degradation of soy proteins and the production of organic acids. We previously reported that heat-killed K040706 enhanced phagocytic ability by upregulating the Toll-like receptor 2 (TLR2)-mediated nuclear factor kappa-light-chain-enhancer of activated B cells (NF-κB) and mitogen-activated protein kinase (MAPK) signaling pathways in macrophages and an immunosuppressed mouse model [10]. Moreover, heat-killed K040706 exerted positive effects on intestinal inflammation and microbiota composition in the dextran sulfate sodium (DSS)-induced colitis mice [11]. Although our previous study showed that heat-killed K040706 increased the indices of immune-related organs in cyclophosphamide (CYP)-induced immunosuppressed mice, the immunostimulatory function and associated molecular mechanism of live K040706 were not clearly identified. Therefore, in this study, we investigated the immunostimulatory activity of live K040706 by focusing on cell-mediated immunity in CYP-induced immunosuppressed mice.

## 2. Materials and Methods

### 2.1. Preparation of Live *Lactobacillus sakei* K040706 (Live K040706)

The strain used in this experiment was *Lactobacillus sakei* K040706 (KCCM11472P) separated as previously reported [10].

### 2.2. Animals

All experiments in the present study were conducted under the university guidelines of an ethical committee for animal care and use of the Kyung Hee University according to an animal protocol (KHUASP(SE)-18-158). Male ICR mice (6 weeks old) weighing 19–23 g were purchased from Orient Bio Inc. (Seongnam-si, Korea) and maintained under constant conditions (temperature: 20 ± 2 °C, humidity: 40–60%, light/dark cycle: 12 h) in the animal room.

### 2.3. CYP-Induced Immunosuppression in Mice

The CYP-induced immunosuppressed model was performed with some modification as described [12,13]. Immunosuppression was induced by the injection of CYP (150 mg/kg/day, intraperitoneal (i.p.)) on days 15, 16, and 17. After a 7 day adaptation period, ICR mice were randomly divided into five groups (*n* = 10): Group 1 (vehicle (0.9% saline), p.o.), Group 2 (CYP, i.p.), Group 3 (CYP + live K040706 10^8^ colony forming unit (CFU)/day, p.o.), Group 4 (CYP + live K040706 10^9^ CFU/day, p.o.), Group 5 (live K040706 10^9^ CFU/day, p.o.). Vehicle and live K040706 (10^8^ CFU or 10^9^ CFU/day) orally administered once a day from 1 to 20 days. All groups were sacrificed at 21 days. The thymus, spleens, Peyer’s patches (PPs), and feces were collected from each mouse for further experiments.

### 2.4. Cell Preparation from Spleen and PPs

Spleens were excised aseptically from sacrificed animals, and splenocytes were isolated from the spleen of mice as previously described [12]. After sacrificing the mice, the small intestines were excised, and visible PPs were carefully isolated from the wall of the small intestine as previously reported [11].

### 2.5. Determination of NK Cytotoxic Activity and Granzyme B Production

NK cytotoxic activity was assessed by evaluating the cytotoxicity of NK cells against YAC-1 cells (NK sensitive-target cell) using an LDH (lactate dehydrogenase) cytotoxicity detection kit (Takata, Singa, Japan). Because YAC-1 is a T cell lymphoma that was induced by inoculation of the Moloney leukemia virus (MLV) into a newborn A/Sn mouse, this cell line is sensitive to the cytotoxic activity of naturally occurring killer cells in mice. YAC-1 cells and splenocytes were prepared with the modified method of previous report [12]. YAC-1 cells were counted, and 5 × 10^5^ cells/well were added to 96-well plates in RPMI 1640 medium. Splenocytes (effector cells) isolated from live K040706-treated mice were activated by IL-2 (20 ng/mL). After treatment with IL-2, the activated splenocytes were added to triplicate wells in a 5:1 ratio (effector-to-target) and were incubated for 4 h. Supernatants from each well (100 μL) were transferred into 96-well plates, and 100 μL of LDH substrate mixture was added. The absorbance of each well at 490 nm was measured using a microplate reader (Molecular Devices Inc., San Jose, CA, USA). The percentage of cytotoxicity was calculated by applying the following formula: NK cell cytotoxicity (%) = ((experimental release − spontaneous release)/(maximum release − spontaneous release)) × 100. Supernatants of reacted YAC-1+ splenocyte cells were measured using a granzyme B kit (DuoSet^®^) (R&D systems, Minneapolis, MN, USA) according to the manufacturer’s instructions. The absorbance at 490 nm was analyzed using a microplate ELISA reader (Molecular Devices Inc.).

### 2.6. Lymphocyte Proliferation Assay

In the CYP-induced immunosuppression model, splenocytes were prepared as described above and seeded at 2 × 10^6^ cells/mL in 96-well plates and then incubated with concanavalin A (Con A, 5 μg/mL for T cell activation) or lipopolysaccharide (LPS, 1 μg/mL for B cell activation) for 48 h. Cell proliferation assay was conducted by using the Cell Titer 96^®^ Aqueous One Solution Reagent (Promega, Madison, WI, USA). After 4 h incubation, the absorbance of each well was measured using a microplate reader (Molecular Devices Inc.) at 490 nm.

### 2.7. Determination of Cytokine Production

Splenocytes and PP cells from CYP-induced immunosuppressed mice were prepared as described above and seeded in 24-well plates. Con A (5 μg/mL) was added to splenocytes. After 48 h incubation, cell culture supernatants were collected and the production of each cytokine was quantified using ELISA kits according to the manufacturer’s instructions (IFN-γ, IL-2, IL-12, IL-10, IL-13, and IL-4, R&D systems: IL-6, BD Pharmingen (San Jose, CA, USA); and granulocyte-macrophage colony-stimulating factor (GM-CSF), BioLegend (San Diego, CA, USA.)

### 2.8. RNA Extraction and Quantitative Real-Time RT-PCR (qRT-PCR)

Total RNA from spleen tissues was extracted using the Easy Blue^®^ kit (Intron Biotechnology, Seoul, Korea). PCR amplification was performed using SYBR Premix Ex Taq (TaKaRa Bio Inc, Shiga, Japan) as described previously [10]. Extracted RNA was reversely transcribed using TOPscript™ RT Dry MIX (Enzynomics, Daejeon, Korea). The oligonucleotide primers listed in Table 1 were designed using Primer3, and the specificity checking module used BLAST. A dissociation curve analysis of *IFN-γ*, *IL-2*, *IL-12*, and *β-actin* showed a single peak for each. PCRs were carried out for 50 cycles under the following conditions: denaturation at 95 °C for 5 s, annealing at 55 °C for 10 s, and elongation at 72 °C for 20 s. The mean Ct values of genes were calculated from triplicate measurements and normalized versus the mean Ct of β-actin.

### 2.9. Analysis of Microbiota

#### 2.9.1. DNA Extraction, Emulsion-Based PCR (emPCR), and Next-Generation Sequencing

The total bacterial genomic DNA of the collected fecal samples was extracted using an *i*-genomic stool plus kit (Intron Biotechnology, Seoul, Korea) according to the manufacturer’s instructions. The library was prepared using PCR products following the GS FLX plus library prep guide. The libraries were quantified using the PicoGreen assay (Victor 3). The emPCR, corresponding to the clonal amplification of the purified library, was carried out using the GS-FLX plus emPCR Kit (454 Life Sciences, Branford, CT USA) as previously reported [11]. After amplification, sequencing was performed on a Genome Sequencer FLX plus (454 Life Sciences, Bradford, PA, USA) by Macrogen Ltd. (Seoul, Korea).

#### 2.9.2. Selection of 16S rRNAs and Taxonomic Assignment

All the sequence reads were compared to the Silva rRNA database using BLAST [11]. The sequence reads with a similar sequence and an E-value less than 0.01 was admitted as a partial 16S rRNA sequence. Non-16S rRNA sequence reads were less than 1%. The taxonomic assignment of the sequenced reads was carried out using the NCBI Taxonomy Database. The five most similar sequences for each sequence read from the database were found by their bit scores and E-values from the BLAST program. The Needleman–Wunsch global alignment algorithm was used to determine the optimum alignment of two sequences along their entire length. Pairwise global alignment was performed on selected candidate hits to find the best-aligned hit. The taxonomy of the sequence with the highest similarity was assigned to the sequence read. By similarity, we assigned the taxonomy down to these taxonomical hierarchies: species with more than 97% similarity, genus 94%, family 90%, order 85%, class 80%, and phylum 75%. Operational taxonomic unit (OTU) CD-HIT-OTU software was used for clustering. The Mothur software was used to analyze microbial communities, and the Shannon–Weaver diversity index and Simpson index were used for species diversity.

### 2.10. Measurement of the Bone Marrow (BM) Cell Proliferation and Cytokine Production on Splenocytes and PP Cells

Male ICR mice (6 weeks old) were orally administered 0.9% saline or aqueous solutions of live K040706 (10^9^ CFU/day) for 20 days. On day 21, after the mice were sacrificed, PP and splenocyte cells were prepared as described above. BM was collected from the femurs of each mouse, and erythrocytes in isolated cell pellets were lysed with Red Blood Cell Lysing Buffer Hybrid-Max^TM^ (Sigma-Aldrich, St. Louis, MO, USA). Pellets washed twice with RPMI 1640 medium were resuspended in RPMI 1640 medium (containing 10% fetal bovine serum (FBS)) and BM cell suspensions were prepared according to a previously reported procedure [14]. Splenocytes and PP cells were prepared and activated with Con A (5 μg/mL) for 48 h. The culture supernatants (50 μL) of splenocytes and PP cells were further cultured with BM cell suspension (1.0 × 10^5^ cells/mL) for 48 h. The numbers of proliferating bone marrow cells and the production of IL-6 and GM-CSF were measured using the Cell Titer 96^®^ Aqueous One Solution Reagent (Promega) and ELISA (Mouse IL-6; (BD Pharmingen) and GM-CSF (BioLegend) methods, respectively.

### 2.11. Statistical Analysis

The results are expressed as the mean ± SD (*n* = 10). Statistically significant values were compared applying assayed using one-way analysis of variance (ANOVA) and the Dunnett’s post hoc test, and *p*-values of less than 0.05 was considered statistically significant.

## 3. Results

### 3.1. Amelioration of Changes in Body Weight and Thymus Index in CYP-Induced Mice by Live K040706

**Figure 1:**
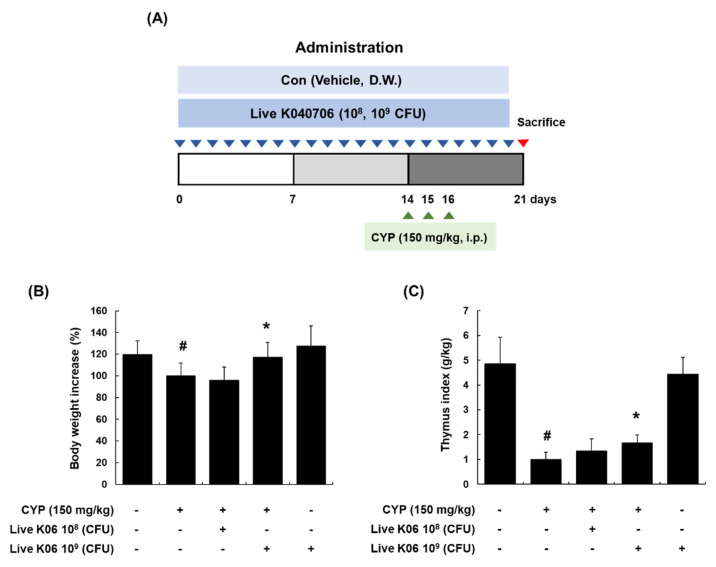
Effects of live K040706 treatment on body weight and thymus index in cyclophosphamide (CYP)-treated mice. (**A**) Schematic diagram of the in vivo experiment. Live K040706 (10^8^ or 10^9^ colony forming unit (CFU)/day) were orally administered for 20 days. (**B**) At 21 days, the mice were weighed, and the change in body weight was calculated. (**C**) Then, the mice were sacrificed, and the thymuses were obtained. The thymus index was calculated with the following formula: thymus index = thymus weight (g)/body weight (kg). Data are presented as the mean ± SD of 10 mice. ^#^ *p* < 0.05 vs. vehicle-treated control mice; * *p* < 0.05 vs. CYP-treated mice.

To evaluate the immunostimulatory activity of live K040706 in CYP-induced immunosuppressed mice, we first investigated whether oral administration of live K040706 (10^8^ or 10^9^ CFU/day) reverses the CYP-induced reduction in body weight and thymus index in mice. The experimental schedule used to establish immunosuppressed mice is shown in Figure 1A. Intraperitoneal injection of one dose of CYP at 150 mg/kg on days 14, 15, and 16 induced a significant reduction in the body weight of mice (*p* < 0.05), which was restored to that of vehicle-treated mice following the administration of high-dose live K040706 (10^9^ CFU/day, p.o.) (Figure 1B). In addition, high-dose live K040706 significantly altered the thymus index (*p* < 0.05, Figure 1C). However, no significant difference was observed in mice treated with a low dose of live K040706 (10^8^ CFU/day, p.o.) (Figure 1B,C).

### 3.2. Live K040706–Mediated Restoration of NK Cell Cytotoxic Activity and Granzyme B Production in CYP-Induced Mice

**Figure 2:**
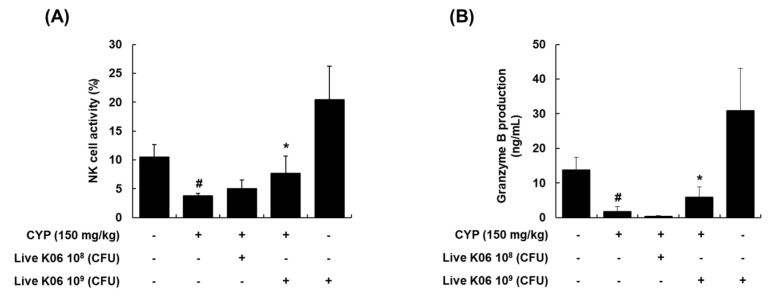
Effects of live K040706 on natural killer (NK) cell activity and granzyme B production in CYP-treated mice. Live K040706 (10^8^ or 10^9^ CFU/day) were orally administered for 20 days. (**A**) Splenocytes from different groups of mice were collected and treated with interleukin (IL)-2 for 72 h. After incubation, YAC-1 cells (target cells) were added to the IL-2-stimulated splenocytes at a ratio of 1:5 (effector-to-target) for 4 h. NK cell cytotoxic activity was measured with an lactate dehydrogenase (LDH) assay. (**B**) Granzyme B production in the cell culture supernatant of reacted YAC-1 cells + splenocytes was determined with ELISA. Data are presented as mean ± SD of 10 mice. ^#^ *p* < 0.05 vs. vehicle-treated control mice; * *p* < 0.05 vs. CYP-treated mice.

NK cells are an important effector of the innate immune system, and when activated by IL-2, the function of NK cells, such as protection against tumor cells and viral infections, is enhanced [15]. Granzyme B, a major component of the granzyme family expressed in NK cells, contributes to target cell lysis mediated by NK cells [16]. NK cells and granzyme B have been recognized as critical immune effectors, and deficiency of these effectors may impair the innate immune defense and surveillance [17]. To determine whether live K040706 has beneficial effects on the regulation of NK cell activity, we evaluated the effects of live K040706 on splenic NK cell cytotoxic activity against YAC-1 target cells using an LDH cytotoxicity detection kit. As shown in Figure 2A, high-dose live K040706 (10^9^ CFU/day, p.o.) treatment induced a significant enhancement (*p* < 0.05) in NK cell activity, which was reduced by 50% or more in the splenocytes isolated from CYP-treated mice. However, there was no significant difference in activity in the CYP + low-dose live K040706 (10^8^ CFU/day, p.o.) group compared with that in the group treated with CYP only. Similarly, a significant recovery was observed in the production of granzyme B in the splenocytes isolated from mice treated with high-dose live K040706 (*p* < 0.05, Figure 2B).

### 3.3. Live K040706–Mediated Increase in Splenocyte Proliferation of CYP-Induced Mice

**Figure 3:**
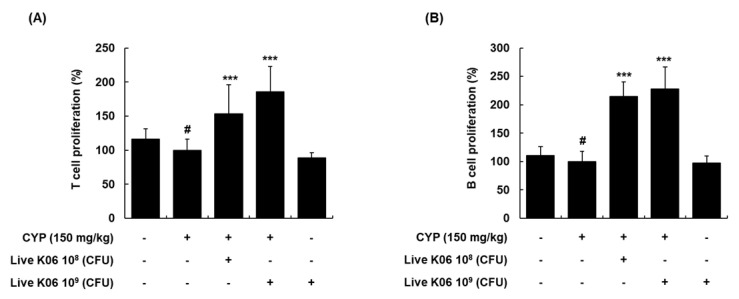
Effects of live K040706 on the proliferation of T and B cells in CYP-treated mice. (**A**,**B**) Splenocytes from different groups of mice were isolated and treated with concanavalin A (Con A) or lipopolysaccharide (LPS) for 48 h. After incubation, cell proliferation was measured with an (3-(4,5-dimethylthiazol-2-yl)-5-(3-carboxymethoxyphenyl)-2-(4-sulfophenyl)-2H-tetrazolium) (MTS) assay. The value for CYP-treated mice was set as 100% cell proliferation. Data are presented as mean ± SD of 10 mice. ^#^ *p* < 0.05 vs. vehicle-treated control mice; *** *p* < 0.001 vs. CYP-treated mice.

In addition to NK cells, the proliferation of T and B cells plays a crucial role in cellular immune responses, and splenocyte proliferation is an indicator of immunostimulation [18]. Therefore, we subsequently evaluated the effects of live K040706 on splenocyte proliferation in CYP-immunosuppressed mice in response to a T-cell-specific mitogen (Con A) and a B-cell-specific mitogen (LPS) [19]. As expected, the proliferative responses of T and B cells were significantly decreased in the CYP-treated group by 15.9% and 10.7%, respectively, compared with those in the vehicle-treated group (Figure 3A,B). However, in the experimental groups, the administration of live K040706 at 10^8^ and 10^9^ CFU/day induced a marked increase in the proliferation of T cells by up to 154% and 185%, respectively (*p* < 0.001, Figure 3A). Moreover, the stimulatory effects of live K040706 (10^8^ or 10^9^ CFU/day) on LPS-induced B cell proliferation were similar to their effects on Con A-induced T cell proliferation (Figure 3B). These results indicated that high- and low-dose live K040706 had a co-mitogenic effect on splenic T and B cells and could affect the strength of the immune response.

### 3.4. Regulation of Th1 Cytokines and Their mRNA Expression in Splenocytes of CYP-Treated Mice by Live K040706

**Figure 4:**
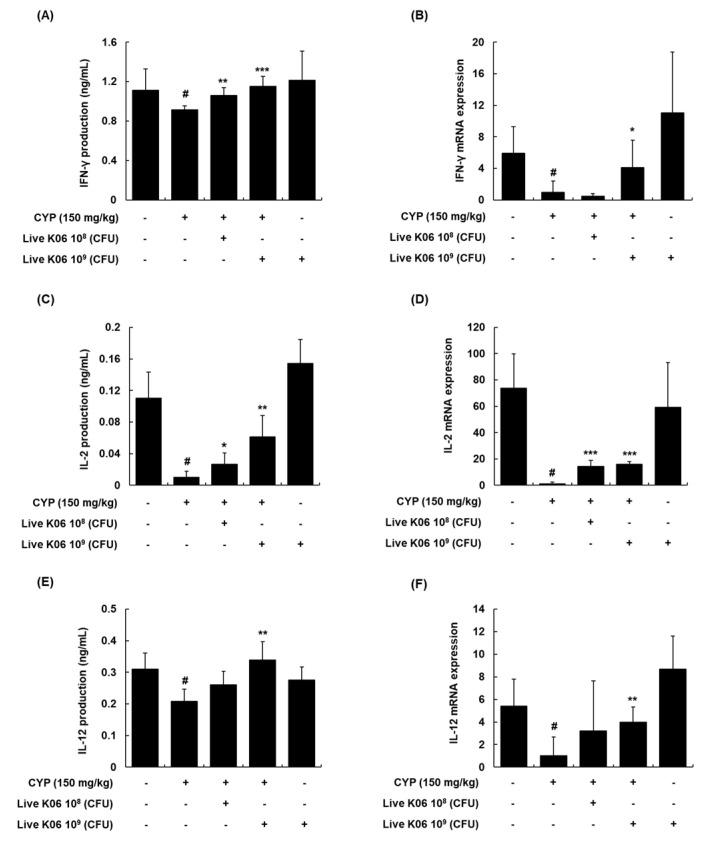
Effects of live K040706 on the production and mRNA expression of interferon (IFN)-γ, IL-2, and IL-12 in CYP-treated mice. (**A**,**C**,**E**) Splenocytes were obtained and treated with Con A for 48 h. After incubation, the culture supernatant was collected and analyzed with ELISA for IL-2, IFN-γ, and tumor necrosis factor (TNF)-α production. (**B**,**D**,**F**) The mRNA expression levels of the cytokines in the splenocytes were determined using qRT-PCR. Data are presented as mean ± SD of 10 mice. ^#^ *p* < 0.05 vs. vehicle-treated control mice; * *p* < 0.05, ** *p* < 0.01, and *** *p* < 0.001 vs. CYP-treated mice.

The sustained production of various cytokines also contributes to protective immunity. Several cytokines, in particular, IFN-γ, IL-2, and IL-12, are mainly secreted by CD4^+^ Th1 cells [20]. In addition, the production of IFN-γ by activated NK cells plays an important role in the initial phase of the innate immune response by inducing the proliferation of monocytes and dendritic cells [21]. IL-2 enhances cellular toxicity, perforin binding, and IFN-γ levels by activating NK cells [22]. IL-12 also stimulates Th1- or cell-mediated immunity by inducing NK cell and T lymphocyte responses [23]. To determine whether live K040706 is involved in the modulation of T helper cells, we first examined the effects of live K040706 on Th1 cell-related cytokines in spleens collected at the end of the experiment. We found that CYP treatment significantly reduced the expression levels of immunoregulatory cytokines in mouse spleen tissues, as demonstrated by the decreased protein and mRNA levels of IFN-γ, IL-2, and IL-12 (*p* < 0.05, Figure 4). The administration of live K040706 induced immune responses in immunosuppressed mice, significantly increasing the splenic protein expression levels of Th1 cell-related cytokines, such as IFN-γ, IL-2, and IL-12 (Figure 4A,C,E). Simultaneously, live K040706 also enhanced *IFN-γ*, *IL-2*, and *IL-12* mRNA expression levels, which were strongly reduced in the presence of CYP (Figure 4B,D,F). Interestingly, live K040706 administration had no significant inductive effect on Th2-related cytokines, such as IL-10 and IL-13, despite the attenuation of CYP-downregulated IL-4 production (Supplementary Materials Figure S1). These findings indicated that the effects of live K040706 on CYP-suppressed cytokine production and mRNA expression were more potent in Th1 than in Th2 cells.

### 3.5. Effects of K040706 on Microbiota Composition in CYP-Treated Mice

**Figure 5:**
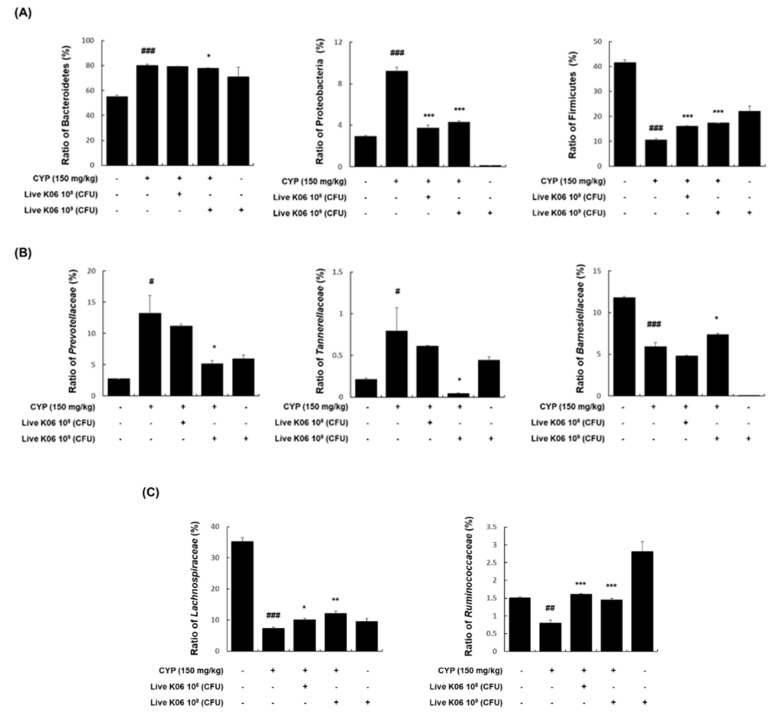
Effects of K040706 treatment on microbiota composition in CYP-treated mice. Genomic DNA was extracted from fecal samples taken from CYP-treated mice or live K040706-treated mice (10^8^ or 10^9^ CFU/day) and analyzed for bacterial composition using 16S rRNA gene sequences. (**A**) The relative abundance of bacterial phyla in fecal samples from CYP-induced mice. (**B**) Relative distribution of families of Bacteroidetes. (**C**) Relative distribution of families of Firmicutes. Data are presented as mean ± SD of 10 mice. ^#^ *p* < 0.05, ^##^ *p* < 0.01, and ^###^ *p* < 0.001 vs. vehicle-treated control mice; * *p* < 0.05, ** *p* < 0.01, and *** *p* < 0.001 vs. CYP-treated mice.

The gut microbiota is known to be an important regulator of normal immune responses, and chemotherapy drugs, such as CYP, can shift the composition of the gut microbiota [24]. With this in mind, we analyzed the effects of live K040706 administration on the gut microbiota composition in CYP-induced immunosuppressed mice. As shown in Figure 5A, the dominant bacterial phyla observed in the mouse colon were Bacteroidetes (55.0% ± 1.3%), Firmicutes (41.5% ± 1.1%), and Proteobacteria (2.9% ± 0.1%). In comparison with the vehicle-treated group, the CYP-treated group showed a high abundance of Bacteroidetes (80.1% ± 1.0%) and Proteobacteria (9.2% ± 0.4%), and a low abundance of Firmicutes (10.5% ± 0.5%). However, both low-dose and high-dose live K040706 supplementation significantly reduced the proportions of Bacteroidetes (79.2% ± 0.1% and 77.8% ± 0.3%, respectively) and Proteobacteria (3.7% ± 0.3% and 4.3% ± 0.1%, respectively) and increased the proportion of Firmicutes (16.2% ± 0.2% and 17.3% ± 0.1%, respectively). Among families of the dominant Bacteroidetes phylum, the abundances of Prevotellaceae and Tannerellaceae were increased in the CYP-treated group (13.2% ± 2.9% and 0.79% ± 0.28%, respectively), but these increases were significantly suppressed by the administration of high-dose live K040706 (5.13% ± 0.51% and 0.04% ± 0.01%). Conversely, the abundance of Barnesiellaceae, which was reduced by CYP (5.9% ± 0.5%), increased significantly following high-dose live K040706 administration (7.37% ± 0.13%) (Figure 5B). Furthermore, among bacterial families of the Firmicutes phylum, the abundances of Lachnospiraceae and Ruminococcaceae were significantly decreased in the CYP-treated group (7.38% ± 0.38% and 0.8% ± 0.08%, respectively), but this reduction was recovered by low-dose (10.08% ± 0.57% and 1.61% ± 0.02%, respectively) and high-dose live K040706 supplementation (12.09% ± 0.8% and 1.45% ± 0.05%, respectively) (Figure 5C).

### 3.6. Promotion of BM Cell Hematopoiesis in Mice by Live K040706

**Figure 6:**
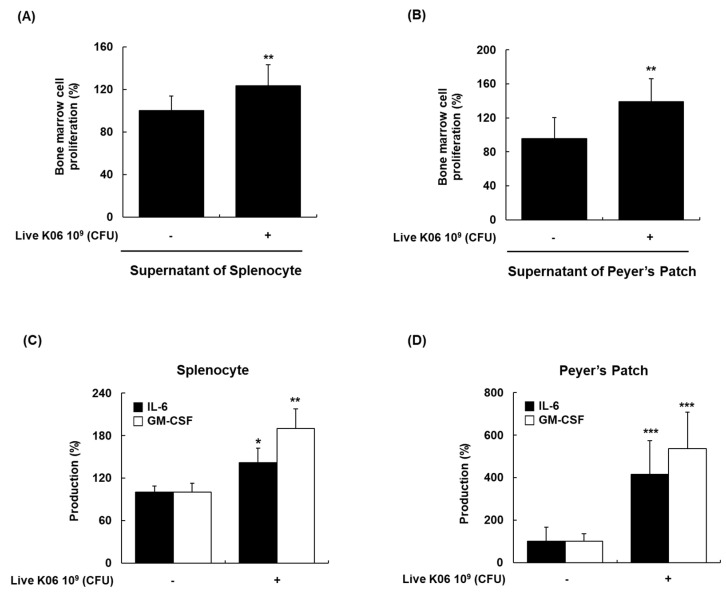
Effects of oral administration of live K040706 on bone marrow (BM) cell proliferation and IL-6 and granulocyte-macrophage colony-stimulating factor (GM-CSF) production. (**A**,**B**) cells were collected from the spleen and PP tissues of live K040706-treated mice (10^9^ CFU/day) or vehicle-treated mice and incubated with Con A for 48 h. The cell culture supernatant was added to BM cells from normal mice and further incubated for 48 h. The proliferation of BM cells was examined using the Cell Titer 96^®^ Aqueous One Solution Reagent. Data are presented as mean ± SD of 10 mice. ** *p* < 0.05 vs. vehicle-treated BM cells. (**C**,**D**) The supernatant was collected and analyzed for IL-6 and GM-CSF production, using ELISA. Data are presented as mean ± SD of 10 mice. * *p* < 0.05, ** *p* < 0.01, and *** *p* < 0.001 vs. vehicle-treated control mice.

The regulation of blood cell formation is vital for the replenishment of mature effector cells of innate and acquired immune responses [25]. Therefore, we examined the effects of live K040706 on hematopoiesis, using mouse BM cells. The cell culture supernatants of splenocytes or PP cells isolated from vehicle-treated or live K040706-treated mice (10^9^ CFU/day) were added to the culture medium of BM cells. The supernatants of splenocytes and PP cells enhanced the proliferation of BM cells by 24% and 39%, respectively (Figure 6A,B). We further investigated the stimulatory effects of live K040706 on hematopoietic regulatory cytokines in the culture supernatants of splenocytes and PP cells. Oral administration of live K040706 (10^9^ CFU/day) significantly increased IL-6 and GM-CSF production in the culture supernatants of both splenocytes and PP cells (Figure 6C,D). These results showed that live K040706 triggered splenocytes and PP cells to produce hematopoietic regulatory factors, leading to BM cell proliferation.

## 4. Discussion

Probiotic products, which consist of live or dead microorganisms, generate a wide range of beneficial biological responses, such as immunostimulatory effects [26]. A number of studies on the immunostimulatory effects of probiotics have been conducted in various fields [27,28]. We previously reported that heat-killed K040706 demonstrated immunostimulatory and anti-inflammatory effects in macrophages in vitro, CYP-induced mice, and DSS-induced colitis mice [10,11]. In the present study, we focused on the immunostimulatory effects of live K040706 in CYP-induced mice and found that live K040706 restored cellular and intestinal immune functions and modulated the intestinal microbiota profile.

CYP is an alkylating drug that induces immunosuppression due to the impairment of immune cells, strongly interfering with the proliferation and differentiation of T and B cells and reducing the number of normal T and B cells [29,30]. Moreover, it leads to weight loss in the thymus, which is involved in the body’s defense mechanism by protecting against various pathogens and inflammatory mediators [31] and modulating the development and differentiation of T cells. Therefore, in the present study, mice treated with CYP were used as an animal model to evaluate the immunostimulatory effects of live K040706. We first assessed the body weight and thymus index of CYP-induced mice. Treatment with CYP (150 mg/kg/day, i.p.) on days 14, 15, and 16 reduced body weight and thymus index, suggesting that an immunosuppressive mouse model was successfully established. Oral administration of high-dose live K040706 (10^9^ CFU/day) alleviated the CYP-induced reduction in body weight and thymus index, suggesting that high-dose live K040706 could maintain body weight and protect the immune organs against CYP-induced immunosuppression.

As splenocytes (consisting of T and B cells, NK cells, and macrophages) are responsible for various immune functions, the proliferation of splenocytes can result in early activation of the humoral and cell-mediated immune enhancement with cytokine expression [32]. Splenocytes are therefore useful for assessing the efficacy of immunostimulatory agents [33]. NK cells are potential lymphocytes of the innate immunity that secrete soluble factors, including cytokines, to activate macrophages and dendritic cells, thus forming the first line of defense against viruses and tumor cells [34]. The activation of NK cells is accompanied by the leakage of granzyme B from intracellular granules into the cytoplasm, causing the apoptosis of target cells [16]. Therefore, assessment of NK cell activity is a valuable method for determining the host’s cellular immune response. NK cell cytotoxicity and granzyme B levels are reduced in immunosuppressed models generated using CYP. To determine whether live K040706 stimulate NK-cell-mediated immune responses, we assessed splenic NK cell activity and granzyme B levels following live K040706 administration in CYP-treated mice. Our results showed that splenic NK cell cytotoxic activity and granzyme B levels were significantly lower in the CYP-treated group than in the vehicle-treated group; these levels were significantly increased by treatment with high-dose live K040706 (10^9^ CFU/day). Considering that CYP can interfere with the proliferation of T and B cells [35,36], a lymphoproliferation assay was used to analyze the effects of probiotics on immune functions. Administration of low-dose (10^8^ CFU/day) or high-dose (10^9^ CFU/day) live K040706 significantly reversed the CYP-induced reduction in splenic T and B cell proliferation. These results indicate that live K040706 can markedly enhance NK cell activity and the proliferation of splenic T and B cells, suggesting that live K040706 could improve cell-mediated immunity and thus could have potential immune activity.

To elucidate the mechanism of the T-cell-mediated immunostimulatory activity of live K040706, we investigated its effects on cytokine production in immunosuppressed mice. Lymphocytes differentiate into Th1 or Th2 cells in response to various effector cytokines; subsequent production of IFN-γ, IL-2, and IL-12 secreted by Th1 cells promotes cell-mediated immunity, whereas IL-4, IL-10, and IL-13 secreted by Th2 cells are involved in humoral immunity [37]. IL-2 is a cytokine essential for the survival and proliferation of T cells, and induces NK cell activation; together, this can restrict the growth and metastases of tumors [17,20]. Administration of high-dose live K040706 significantly increased IFN-γ, IL-2, and IL-12 production and their mRNA expression, which were reduced by CYP. Accordingly, increased IL-2 secretion may stimulate T cell proliferation and IFN-γ production, which in turn can improve the immune response by enhancing cellular immunity against cancer and pathogen-infected cells. We also found that the administration of high-dose live K040706 significantly restored IFN-γ, IL-2, and IL-12 production in the PP cells of CYP-induced immunosuppressed mice (Supplementary Materials Figure S2). In addition, we found that live K040706 significantly increased IL-4 production in the splenocytes of immunosuppressed mice, while they did not have any effect on IL-10 and IL-13 production. These results indicate that live K040706 could enhance the cellular immune response by upregulating Th1 cytokines rather than Th2 cytokines. Therefore, K040706 may benefit the host by activating defense mechanisms against intracellular pathogens, such as viruses and bacterial infections.

The microbiota plays an important role in physiological functions, such as modulation of the immune system [26]. Therefore, to confirm whether live K040706 protect the host’s immune system, we analyzed the microbial composition of CYP-induced mice. The gut microbiota of mice is composed of various microbes residing on the mucosal surface, with a dominant distribution of Bacteroidetes, Firmicutes, and Proteobacteria. CYP treatment increased the abundance of Bacteroidetes while decreasing that of Firmicutes. However, treatment with live K040706 slightly restored the levels of Bacteroidetes, Firmicutes, and Proteobacteria to the levels seen prior to CYP treatment. Proteobacteria is the largest bacterial phylum, which includes various pathogenic bacteria, such as *Escherichia coli*, *Salmonella*, *Vibrio cholerae*, and *Helicobacter pylori* [38]. The chronic enrichment of Proteobacteria in the gut may represent an imbalanced, unstable microbial community structure or a state of disease of the host [39]. Previously, CYP treatment was found to induce the expansion of the Proteobacteria population [40]. In this study, the administration of live K040706 prevented an increase in the abundance of Proteobacteria. These results indicate that live K040706 may mitigate immune-driven dysbiosis in CYP-induced immunosuppressed mice.

We subsequently investigated the prevalence of Prevotellaceae, Barnesiellaceae, Tannerellaceae, Lachnospiraceae, and Ruminococcaceae to identify specific phylotypes belonging to Bacteroidetes and Firmicutes at the family level. CYP has been reported to increase the abundance of Ruminococcaceae, but decrease the abundance of Prevotellaceae and Lachnospiraceae [41]. However, the composition of Barnesiellaceae and Tannerellaceae in the gut microbiota following CYP administration remains unknown. In our study, the abundance of Prevotellaceae, Tannerellaceae, and Ruminococcaceae was increased by CYP but decreased after oral administration of live K040706. On the other hand, the reduced abundance of Barnesiellaceae in CYP-induced mice was slightly increased by the administration of live K040706; however, the abundance of Lachnospiraceae remained unchanged. Other studies have demonstrated that the enrichment of Barnesiellaceae is important for protection against bloodstream infection [42] and that the family Tannerellaceae is most abundant in individuals with gastrointestinal disorders, such as Crohn’s disease (CD) [43]. Additionally, Ruminococcaceae has been reported to be increased in colonic CD patients [44] and ankylosing spondylitis patients [45]. These findings are consistent with our results, which show that the administration of live K040706 may play an important role in improving host immunity by balancing the microbiota composition altered by CYP. However, it was unclear whether the change in the microbiota profile was crucial for the immunostimulatory effects of live K040706 or if there was any correlation between them. Further studies are needed to determine the relationship between changes in the microbiota and immune responses, in order to clarify the mechanism of the immunostimulatory activity of K040706. Live K040706 administration without CYP treatment markedly increased NK cell activity and changed the microbiota composition, such as altering Bacteroidetes and Firmicutes levels, indicating that K040706 itself possesses immunomodulatory properties. Despite many studies, the causal relationship between changes in the microbiota and immunosuppression by CYP and the major bacteria involved have not yet been identified. Under these circumstances, it is difficult to accurately interpret microbiota changes in live K040706 and/or CYP. The analyses of microbiota data of this manuscript were presented that microbiota distribution changed by K040706 and/or CYP can be the basis for finding the link between microbiota and immune regulation. Further in-depth research is needed to provide an accurate link between the change in microbiota caused by live K040706 and its immunostimulating effects.

Hematopoiesis is the process by which blood and immune cells of distinct lineages (T and B lymphocytes, NK cells, and macrophages) are produced according to the migration of hematopoietic stem cells (HSCs) within the BM microenvironment [46]. Hematopoietic homeostasis disruption caused by various factors, including anticancer therapies, myeloid malignancies, and nutritional deficiencies, may lead to the development of myelosuppression; thus, the enhancement of hematopoiesis may be beneficial for the immune response [46,47]. Administration of K040706 increased the proliferation of BM cells in normal mice and promoted the secretion of GM-CSF by splenocytes and PP cells. GM-CSF is known to contribute to hematopoietic restoration by strengthening the survival of HSCs and the proliferation and differentiation of distinct lineage cells [48]. IL-6 is particularly important in modulating the differentiation and proliferation of HSCs, and is required for macrophage colony-stimulating factor (M-CSF) and granulocyte-colony stimulating factor (G-CSF) activity during hematopoiesis [48]. Various immunostimulatory agents can increase BM cell proliferation and promote an increase in cytokines associated with BM cell proliferation. In this study, we showed that live K040706 directly activated splenocytes and PP cells to produce hematopoietic cytokines (IL-6 and GM-CSF), which was sufficient to induce the proliferation of mouse BM cells. Although we additionally assessed the production of M-CSF, IL-3, and IL-7 to evaluate the other factors affecting hematopoietic regulation, the oral administration of live K040706 (10^9^ CFU/day) did not significantly affect M-CSF, IL-3, or IL-7 production in the supernatants of either splenocytes or PP cells (data not shown). Kevin et al. reported that TLR2 activation specifically induces the expression of IL-6 and GM-CSF genes, whereas there was no significant induction of IL-3, IL-7, and M-CSF in stimulated Human umbilical vein endothelial cells (HUVECs) [49]. Similar to these results, our previous study showed that live K040706 improved the immune function by regulating immunological parameters, such as IL-6 production, and by activating TLR2 in rIFN-γ-primed macrophages [10]. Since live K040706 act as a TLR2 agonist, it is believed that the primary target genes of live K040706 are IL-6 and GM-CSF, instead of IL-3, IL-7, and M-CSF. Considering these results, we suggest that live K040706-induced IL-6 and GM-CSF production plays a more important role in bone marrow cell proliferation involving hematopoietic regulation. Taken together, our results indicate that the immunostimulatory activity of live K040706, mediated by an increase in cytokines, has positive effects on hematopoiesis and the production of immune cells.

## 5. Conclusions

In summary, the current study demonstrates that oral administration of live K040706 ameliorated CYP-suppressed cellular immunity by increasing the thymus index, T and B cell proliferation, NK cell activity, and Th1 cytokine production. The immunostimulating effects of live K040706 may be associated with a shift in the microbiota composition toward a normal profile. Furthermore, live K040706 stimulated the proliferation of BM cells through IL-6 and GM-CSF upregulation. Therefore, K040706 may be used as a potential dietary supplement, which could be beneficial for preventing chemotherapy-induced immunosuppression or maintaining a healthy immune system.

## Figures and Tables

**Table 1:** The list of primer sequences for qRT-PCR.

Gene	Forward	Reverse	Tm (°C)
*IFN-γ*	GCTTCCTGAGGCTGGATTC	TACCTTCTTCAGCAACAGCAAG	55
*IL-2*	TCCTGGGGAGTTTCAGGTTC	CTCTACAGCGGAAGCACAGC	55
*IL-12*	TCTGCAGAGAAGGTCACACT	ATGAAGAAGCTGGTGCTGTA	55
*β-actin*	ATCACTATTGGCAACGAGCG	TCAGCAATGCCTGGGTACAT	55

## Supplementary Materials

The following are available online at https://www.mdpi.com/2072-6643/12/11/3573/s1. Figure S1: Effects of live K040706 on the production of IL-4, IL-10, and IL-13 in splenocytes of CYP-treated mice. Figure S2: Effects of live K040706 on the production of IFN-γ, IL-2, and IL-12 in Peyer’s patch of CYP-treated mice.
