# Supplementary material for: Immunostimulatory Effects of Live Lactobacillus sakei K040706 on the CYP-Induced Immunosuppression Mouse Model
Source: Nutrients. 2020 Nov 22;12(11):3573. doi: 10.3390/nu12113573 (PMC7700367; doi:10.3390/nu12113573)
Supplement: Supplementary file 1 [file nutrients-12-03573-s001.pdf]

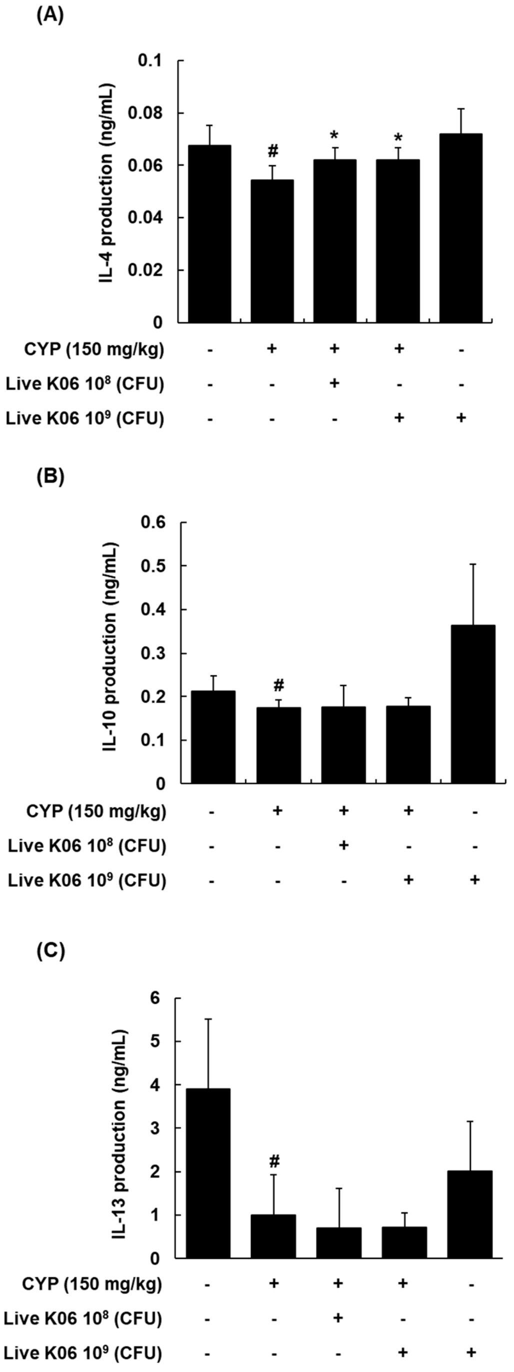

Figure S1. Effects of live K040706 on the production of IL-4, IL-10, and IL-13 in splenocytes of CYP-treated mice. (**A-C**) Splenocytes were obtained and treated with Con A for 48 h. After incubation, the culture supernatant was collected and analyzed for IL-4, IL-10, and Il-13 production by ELISA. Data are presented as the mean ± SD of 10 mice. $^{\#}p < 0.05$ vs vehicle-treated mice; $^{*}p < 0.05$ vs CYP -treated mice.

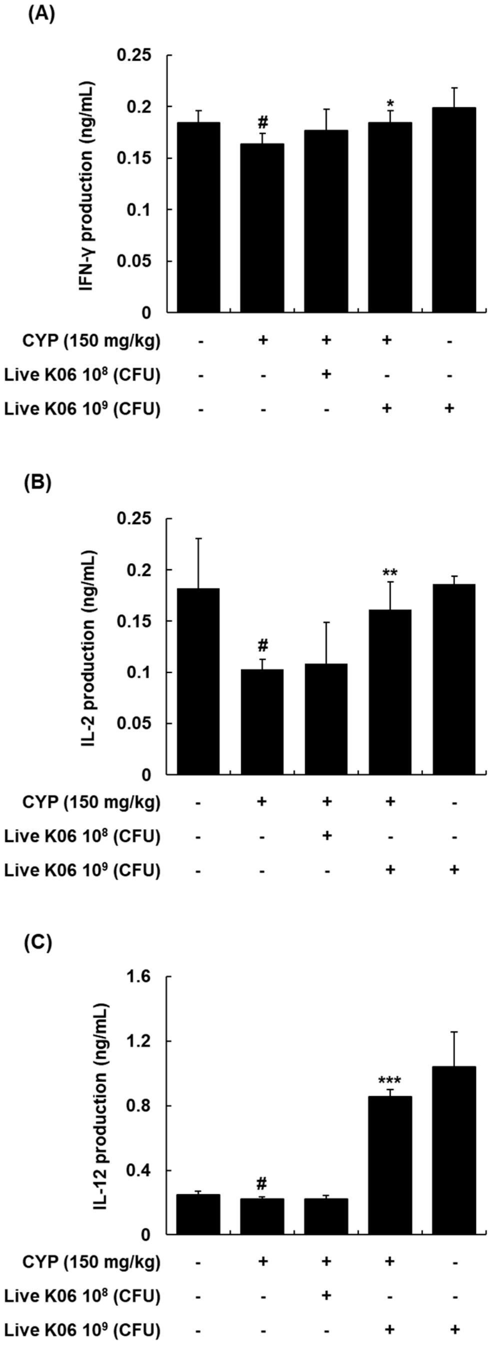

Figure S2. Effects of live K040706 on the production of IFN-γ, IL-2, and IL-12 in Peyer's patch of CYP-treated mice. (**A-C**) Isolated Peyer's patche cells were obtained and treated with Con A for 48 h. After incubation, the culture supernatant was collected and analyzed for IFN-γ, IL-2, and IL-12 production by ELISA. Data are presented as the mean ± SD of 10 mice. $^{\#}p < 0.05$ vs vehicle-treated mice; $^{*}p < 0.05$, $^{**}p < 0.01$, and $^{***}p < 0.001$ vs CYP -treated mice.
